# Cancer risk following pernicious anaemia.

**DOI:** 10.1038/bjc.1989.169

**Published:** 1989-05

**Authors:** L. A. Brinton, G. Gridley, Z. Hrubec, R. Hoover, J. F. Fraumeni

**Affiliations:** Division of Cancer Etiology, National Cancer Institute, Bethesda, MD 20892.

## Abstract

A computer-based file of all Veterans Administration (VA) hospitalisation records for the period 1969-1985 was used to identify and follow for cancer development a cohort of 5,161 white males with pernicious anaemia. A total of 34,915 person-years were accrued, with an average length of follow-up of 6.8 years. A total of 481 cancers were diagnosed, slightly higher than the number expected (SIR = 1.2). Significant excesses were observed for cancers of the buccal cavity and pharynx (1.8) and stomach (3.2), and for melanoma (2.1), multiple myeloma (2.1), myeloid leukaemia (3.7) and other and unspecified leukaemia (4.0). Although the excess for stomach cancer was highest in the first year after diagnosis in a VA hospital, risks of 2-fold or greater persisted throughout the study period. The majority of leukaemias occurred in the first year of follow-up, but some excess risk continued beyond this time. The elevated risk of buccal and pharyngeal cancers may relate to heavy alcohol intake among this population, although risks remained high even when the cohort was restricted to patients without an admission for alcoholism. Although an elevated risk of stomach cancer among pernicious anaemia patients is consistent with most previous surveys, the low absolute risk suggests that the cost-effectiveness of intensive screening should be reassessed.


Br. J. Cancer (1989), 59, 810–813 © The Macmillan Press Ltd., 1989

### Cancer risk following pernicious anaemia

L.A. Brinton, G. Gridley, Z. Hrubec, R. Hoover & J.F. Fraumeni, Jr

*Epidemiology and Biostatistics Program , Division of Cancer Etiology, National Cancer Institute; Executive Plaza North, Room 443; Bethesda, MD 20892, USA.*

**Summary** A computer-based file of all Veterans Administration (VA) hospitalisation records for the period 1969–1985 was used to identify and follow for cancer development a cohort of 5,161 white males with pernicious anaemia. A total of 34,915 person-years were accrued, with an average length of follow-up of 6.8 years. A total of 481 cancers were diagnosed, slightly higher than the number expected (SIR = 1.2). Significant excesses were observed for cancers of the buccal cavity and pharynx (1.8) and stomach (3.2), and for melanoma (2.1), multiple myeloma (2.1), myeloid leukaemia (3.7) and other and unspecified leukaemia (4.0). Although the excess for stomach cancer was highest in the first year after diagnosis in a VA hospital, risks of 2-fold or greater persisted throughout the study period. The majority of leukaemias occurred in the first year of follow-up, but some excess risk continued beyond this time. The elevated risk of buccal and pharyngeal cancers may relate to heavy alcohol intake among this population, although risks remained high even when the cohort was restricted to patients without an admission for alcoholism. Although an elevated risk of stomach cancer among pernicious anaemia patients is consistent with most previous surveys, the low absolute risk suggests that the cost-effectiveness of intensive screening should be reassessed.

An excess risk of stomach cancer following the diagnosis of pernicious anaemia has been documented for many years, consistent with the underlying atrophic gastritis and histamine-resistant achlorhydria. It has been estimated that the risk of stomach cancer is increased 3- to 6-fold in pernicious anaemia patients (Berkson *et al.*, 1956; Elsborg & Mosbech, 1979; Eriksson *et al.*, 1981; Hitchcock *et al.*, 1955; Kaplan & Rigler, 1945; Zamcheck *et al.*, 1955), but such figures derive from autopsy studies or prevalence surveys which may have inherent sources of bias. In one prospective study (Blackburn *et al.*, 1968), deaths due to stomach cancer were elevated 4-fold, with evidence of continued elevations 10 or more years after initial treatment, but comparable information on cancer incidence was unavailable.

To clarify the effects of pernicious anaemia on subsequent cancer incidence and to assist in formulating policies for screening these patients, we performed a retrospective cohort analysis of patients diagnosed with pernicious anaemia during 1969–1985 in all Veterans Administration hospitals. The large size of the study population allowed assessment of stomach cancer risk according to several variables, including interval since diagnosis of pernicious anaemia. In addition, the study enabled evaluation of the risk of other cancers, notably leukaemia, which may be linked to pernicious anaemia (Corcino *et al.*, 1971; Nielsen & Jensen, 1970; Zarafonetis *et al.*, 1975).

### Methods

The study cohort was defined from patients hospitalised at Veteran Administration institutions across the USA, estimated as representing at least 12% of all hospitalisations among male veterans (Geoghegan & Page, 1984). A total of 7,524 patients aged 18 years and older with pernicious anaemia (diagnostic code 281.0 in the 9th revision of the ICD) were selected from the computer-based file of Veterans Administration (VA) hospitalisation records for 1 July 1969 to 30 September 1985 (4,364,184 veterans). Person-years were accrued starting the day after hospital discharge for the first reported diagnosis of pernicious anaemia in the VA medical record, and subjects were followed until the date of first cancer, date of death, age 100, or 30 September 1985, whichever occurred first. Altogether 2,361 (31.4%) of the 7,524 pernicious anaemia patients were excluded from this analysis for the following reasons (in order of exclusion): died on index admission (495), non-whites (832), females (109) and cancer diagnosed before or same time as first diagnosis of pernicious anaemia (925).

Correspondence: L.A. Brinton.

Rates of the cancers observed at different sites were computed for the remaining 5,761 white, male pernicious anaemia patients. Patients were assumed to be alive and free from cancer unless data in the VA hospitalisation file indicated otherwise. Cancer was defined as codes 140–172, 175, 185–208 of the 9th revision of the ICD (US Public Health Service, 1980).

Expected cancer rates were computed for the four million VA patients in the entire file, following the same exclusion criteria as listed above, except that the person years were calculated from the date of the first VA hospital discharge, rather than from the date of the first hospital discharge diagnosis of pernicious anaemia.

Standardised incidence ratios (SIRs), the ratio of observed to expected number of cancers, were computed by 5-year age groups and 5-year calendar time periods using a life table approach. Confidence intervals were computed by the exact procedure of Liddell (1984).

### Results

The 5,161 patients with pernicious anaemia contributed 34,915 person-years of follow-up (Table I). The mean age at study entry was 67.6 years and the mean follow-up period was 6.8 years. The average year of study entry was 1977, while the mean year of cancer diagnosis was 1979.

Table II presents observed *vs.* expected numbers for all cancers combined and for individual cancer sites. For all sites, the observed number of 481 was slightly higher than the expectation of 415.8, resulting in an SIR of 1.2 (95% CI 1.1–1.3). Significant excesses were observed for several sites, including cancers of the tongue (SIR = 2.2, 11 cases), hypopharynx (SIR = 2.9, 8 cases), pharynx otherwise unspecified

**Table I** Characteristics of men diagnosed with pernicious anaemia in Veterans Administration hospitals, 1969–1985

| | |
|---|---|
| Number of men identified from admission records | 5,161 |
| Person years of follow-up | 34,915 |
| Mean years of follow-up | 6.8 |
| Mean age at study entry | 67.6 |
| Mean year of study entry | 1977.1 |
| Mean age at end of follow-up | 72.8 |
| Mean year of cancer diagnosis | 1978.8 |

(SIR = 4.0, 5 cases), stomach (SIR = 3.2, 31 cases) and for melanoma (SIR = 2.1, 12 cases), multiple myeloma (SIR = 2.1, 9 cases), myeloid leukaemia (SIR = 3.7, 17 cases) and other and unspecified leukaemia (SIR = 4.0, 9 cases). Non-significant excesses of approximately 2-fold (based on at least three observed events) were observed for cancers of the oropharynx (SIR = 1.8, 6 cases), biliary tract (SIR = 1.9, 4 cases) and bone (SIR = 2.3, 6 cases). A total of 80 lung cancers occurred among the cohort, a risk lower than expectation (SIR = 0.8). Other major cancers had observed numbers close to expectation, as did cancers of ill-defined and non-specified sites.

To explore more extensively cancer risks that significantly deviated from expectation, SIRs were examined according to the interval from pernicious anaemia to development of cancer (Table III). For stomach cancer, the largest excess risk (SIR = 7.6) was in the year after first admission for

**Table II** Standardised incidence ratios of observed cancers following the diagnosis of pernicious anaemia, compared to all patients treated at Veterans Administration hospitals

| *Cancer site (ICD-9 code)* | *Observed numbers* | *Expected numbers* | *SIR* | *95% CI* |
|---|---|---|---|---|
| All cancers | 481 | 415.75 | 1.16 | (1.1–1.3)[a] |
| Lip (140) | 8 | 6.62 | 1.21 | (0.5–2.4) |
| Tongue (141) | 11 | 5.09 | 2.16 | (1.1–3.9)[a] |
| Salivary gland (142) | 2 | 1.46 | 1.37 | (0.2–5.0) |
| Gum (143) | 2 | 0.62 | 3.23 | (0.4–11.7) |
| Floor of mouth (144) | 4 | 3.32 | 1.21 | (0.3–3.1) |
| Other parts of mouth (145) | 4 | 3.45 | 1.16 | (0.3–3.0) |
| Oropharynx (146) | 6 | 3.25 | 1.84 | (0.7–4.0) |
| Hypopharynx (148) | 8 | 2.75 | 2.91 | (1.2–5.7)[a] |
| Unspecified pharynx (149) | 5 | 1.25 | 4.00 | (1.3–9.3)[a] |
| Oesophagus (150) | 11 | 7.49 | 1.47 | (0.7–2.6) |
| Stomach (151) | 31 | 9.65 | 3.21 | (2.2–4.6)[a] |
| Small intestine (152) | 1 | 0.77 | 1.30 | (0.0–7.2) |
| Colon (153) | 34 | 32.50 | 1.05 | (0.7–1.5) |
| Rectum and rectosigmoid junction (154) | 14 | 14.72 | 0.95 | (0.5–1.6) |
| Liver (155) | 2 | 3.84 | 0.52 | (0.1–1.9) |
| Biliary tract (156) | 4 | 2.07 | 1.93 | (0.5–4.9) |
| Pancreas (157) | 7 | 9.68 | 0.72 | (0.3–1.5) |
| Other digestive (159) | 2 | 0.62 | 3.25 | (0.4–11.7) |
| Nasal cavities and sinuses (160) | 2 | 3.25 | 0.62 | (0.1–2.2) |
| Larynx (161) | 11 | 11.73 | 0.94 | (0.5–1.7) |
| Lung (162) | 80 | 99.67 | 0.80 | (0.6–1.0) |
| Bone (170) | 6 | 2.60 | 2.31 | (0.8–5.0) |
| Connective and other soft tissue (171) | 5 | 5.37 | 0.93 | (0.3–2.2) |
| Melanoma (172) | 12 | 5.71 | 2.10 | (1.1–3.7)[a] |
| Breast (175) | 1 | 0.83 | 1.20 | (0.0–6.7) |
| Prostate (185) | 90 | 82.98 | 1.08 | (0.9–1.3) |
| Testis and other male genital (186, 187) | 3 | 1.74 | 1.72 | (0.4–5.0) |
| Bladder (188) | 24 | 28.19 | 0.85 | (0.6–1.3) |
| Kidney and other urinary tract (189) | 10 | 7.88 | 1.27 | (0.6–2.3) |
| Eye (190) | 1 | 1.65 | 0.61 | (0.0–3.4) |
| Brain (191) | 5 | 3.70 | 1.35 | (0.4–3.2) |
| Other parts of nervous system (192) | 1 | 1.16 | 0.87 | (0.0–4.8) |
| Thyroid (193) | 1 | 0.88 | 1.14 | (0.0–6.3) |
| Other endocrine (194) | 1 | 0.57 | 1.77 | (0.0–9.8) |
| Ill-defined site (195) | 10 | 10.76 | 0.93 | (0.4–1.7) |
| Unspecified site (199) | 10 | 8.56 | 1.17 | (0.6–2.2) |
| Lymphosarcoma and reticulum cell sarcoma (200) | 5 | 3.69 | 1.36 | (0.4–3.2) |
| Hodgkin's disease (201) | 1 | 1.33 | 0.75 | (0.0–4.2) |
| Other neoplasm of lymphoid tissue (202) | 6 | 5.05 | 1.19 | (0.4–2.6) |
| Multiple myeloma (203) | 9 | 4.30 | 2.08 | (1.0–4.0)[a] |
| Lymphatic leukaemia (204) | 4 | 5.22 | 0.77 | (0.2–2.0) |
| Myeloid leukaemia (205) | 17 | 4.65 | 3.66 | (2.1–5.9)[a] |
| Monocytic leukaemia (206) | 1 | 0.44 | 2.27 | (0.0–12.7) |
| Other and unspecified leukaemia (207, 208) | 9 | 2.23 | 4.03 | (1.8–7.7)[a] |

[a]95% CI excludes 1.0.

**Table III** Standardised incidence ratios of selected cancers by years since first admission for pernicious anaemia, compared to all patients treated at ~~Veterans~~ Administration hospitals

| *Cancer site (ICD-9 code)* | *Years since admission for pernicious anaemia* 1 | 2–5 | 6+ |
|---|---|---|---|
| Stomach (151) | | | |
| Observed number | 12 | 10 | 9 |
| Expected number | 1.59 | 4.36 | 3.69 |
| O/E | 7.55 | 2.29 | 2.44 |
| 95% CI | 3.9–13.2 | 1.1–4.2 | 1.1–4.6 |
| Buccal cavity and pharynx (140–149) | | | |
| Observed number | 6 | 22 | 22 |
| Expected number | 4.76 | 13.04 | 10.67 |
| O/E | 1.26 | 1.68 | 2.06 |
| 95% CI | 0.5–2.7 | 1.1–2.6 | 1.3–3.1 |
| Melanoma (172) | | | |
| Observed number | 2 | 6 | 4 |
| Expected number | 0.93 | 2.54 | 2.23 |
| O/E | 2.14 | 2.36 | 1.79 |
| 95% CI | 0.3–7.8 | 0.9–5.1 | 0.5–4.6 |
| Multiple myeloma (203) | | | |
| Observed number | 1 | 4 | 4 |
| Expected number | 0.66 | 1.86 | 1.80 |
| O/E | 1.52 | 2.15 | 2.22 |
| 95% CI | 0.0–8.4 | 0.6–5.5 | 0.6–5.7 |
| Myeloid leukaemia (205) | | | |
| Observed number | 10 | 3 | 4 |
| Expected number | 0.74 | 2.07 | 1.84 |
| O/E | 13.51 | 1.45 | 2.17 |
| 95% CI | 6.5–24.9 | 0.3–4.2 | 0.6–5.6 |
| Other and unspecified leukaemia (207, 208) | | | |
| Observed number | 5 | 4 | 0 |
| Expected number | 0.36 | 1.02 | 0.85 |
| O/E | 13.89 | 3.92 | 0 |
| 95% CI | 4.5–32.4 | 1.1–10.0 | 0.0–4.3 |

pernicious anaemia, but risks remained elevated during the next 4-year period (SIR = 2.3) as well as later (SIR = 2.4). The risk of cancers of the buccal cavity and pharynx increased steadily following diagnosis of pernicious anaemia, being 1.3 in the first year, 1.7 in the 2–5 year category and 2.1 in the 6+ year period. The risks of melanoma and multiple myeloma were elevated across all time periods, while the leukaemia excesses predominated in the period soon after diagnosis of pernicious anaemia. Although 10 of the 17 observed cases of myeloid leukaemia and five of the nine other and unspecified leukaemia occurred during the first year of follow-up, some excess risk continued to persist beyond this time.

Because of concerns that heavy alcohol intake might have affected some of the observed risks, the cohort was divided into subjects with at least one mention of an admission for alcoholism or alcohol-related diagnosis (alcoholic psychosis, cirrhosis of liver, toxic effect of alcohol) ($n = 865$) and those with no such diagnoses ($n = 4,296$). Although the stomach cancer risk remained elevated among the non-alcoholics (SIR = 3.3, 95% CI 2.2–4.8), the risk associated with cancers of the buccal cavity and pharynx was reduced from 1.8 to 1.5, an excess of borderline significance (95% CI 1.0–2.0). The latency effect previously observed for cancers of the buccal cavity and pharynx did not persist, with the SIRs being 1.3, 1.7 and 1.3, respectively, for the three time periods examined in Table III.

### Discussion

Our findings indicated an excess risk of stomach cancer among patients with pernicious anaemia. Although the risk was highest soon after the diagnosis of pernicious anaemia (SIR = 7.6 for cancers developing within the first year), elevated risks of about 2-fold persisted after this time.

The risk estimates of this study are consistent with most prior investigations. Among 877 patients reported with gastric cancer to the Danish Cancer Registry, Elsborg and Mosbech (1979) found a higher prevalence of pernicious anaemia (2.2%) than in an equal number of colon cancer controls (0.3%). However, the prevalence in the control group was considerably lower than expected based on other surveys, and the interval between the diagnosis of pernicious anaemia and gastric cancer was short in many cases. It was estimated that the risk of gastric cancer among pernicious anaemia patients was approximately three times higher than expected. Similar conclusions were drawn by Eriksson *et al.* (1981) in a prevalence study based on autopsy-verified stomach cancer. Although the 2.1% prevalence of pernicious anaemia among 917 gastric cancer patients was not significantly higher than that of 1.4% among age-matched controls, the difference was significant when analysis was restricted to patients diagnosed with pernicious anaemia more than 5 years before cancer diagnosis. In addition, a follow-up study of 1,625 pernicious anaemia patients (Blackburn *et al.*, 1968) found that, after excluding deaths in early periods when selective inclusion of patients may have occurred, the 29 deaths from stomach cancer significantly exceeded the 7.3 deaths expected, resulting in a SIR of 4.0 – a figure only slightly higher than the risk of 3.2 found in the present study.

An unexpected finding in our study was the excess risk for cancers of the buccal cavity and pharynx. Risk was significantly elevated for cancers of the tongue (SIR = 2.2), hypopharynx (2.9) and pharynx otherwise unspecified (4.0), while a non-significant risk of 1.8 was observed for cancer of the oropharynx. In addition, when examined as a group, the risk of oral and pharyngeal cancers increased with the interval since diagnosis of pernicious anaemia, reaching a significant risk of 2-fold with 6 or more years of latency. The major risk factors for oral and pharyngeal cancers are smoking and drinking (Blot *et al.*, 1988). However, since the risks for lung cancer were low in our survey (SIR = 0.8), it appeared that smoking may be less prevalent than in the general population. The role of alcohol intake cannot be discounted since the risks of oral and pharyngeal cancer were reduced when the analysis was restricted to individuals without a previous admission for alcoholism. The trend towards increasing risks with intervals since diagnosis of pernicious anaemia is intriguing, although it did not persist in individuals without a previous diagnosis of alcoholism. Since dietary micronutrients may play a protective role in oral and pharyngeal cancer (McLaughlin *et al.*, 1988), it is possible that cobalamin (vitamin $B_{12}$) deficiency contributes to the risk, as may the dysplastic changes of the buccal cavity associated with pernicious anaemia (Mitchell *et al.*, 1986).

Haematopoietic malignancies have been repeatedly observed in case reports of pernicious anaemia, including myeloid leukaemia (Blackburn *et al.*, 1968; Corcino *et al.*, 1971; Nielsen & Jensen, 1970; Zarafonetis *et al.*, 1975), polycythaemia vera (Zarafonetis *et al.*, 1975; Mitchell *et al.*, 1986; Engel & Stickney, 1962; Riddle & Davidson, 1968), and multiple myeloma (Fraser, 1969; Larsson, 1962). Thus, it is noteworthy that we observed significant excesses of multiple myeloma (SIR = 2.1), myeloid leukaemia (3.7), and other unspecified leukaemia (4.0). Ethnic and geographic factors may play a role, since rates for both pernicious anaemia and multiple myeloma are elevated in people of northern European origin and in residents of the north central part of the United States (Mason *et al.*, 1981; Blattner *et al.*, 1981). Furthermore, the cohort may have mistakenly included some myelodysplastic or preleukaemic states that can exhibit erythroid hyperplasia with megalobastosis in the marrow (Chanarin, 1969). This explanation is consistent with the predominance of leukaemia in the first year of follow-up, although the persistent excess in risk suggests that a true relationship may exist. If so, the risk may be influenced by the presence of immune alterations, including monoclonal immunoglobulinaemia, as well as chromosome abnormalities in marrow cells of patients with pernicious anaemia (Burnier *et al.*, 1976; Lowe, 1976). Thus, the declining risk of leukaemia with time may result from vitamin $B_{12}$ therapy and its reversal of immunological, cytogenetic or other mechanisms that may affect the leukaemic process. In a clinical report of acute non-lymphocytic leukaemia with pernicious anaemia, treatment with vitamin $B_{12}$ appeared to enhance the differentiation of leukaemic cells (Vogelsang & Spivak, 1984).

The elevated risk of melanoma, which persisted across all follow-up periods, was unanticipated. However, it may be due to the predisposition of fair-complexioned people of northern European descent to both diseases, to diagnostic surveillance of patients with pernicious anaemia, or to the association with immunodeficiency which increases the risk of melanoma, as seen in kidney transplant recipients (Greene *et al.*, 1981).

In interpreting our results, several methodological issues warrant attention. The inclusion of patients in this survey relied on the mention of pernicious anaemia as a discharge diagnosis, possibly resulting in some diagnostic misclassification. However, the fact that all patients were ascertained during a period when standard diagnostic criteria for pernicious anaemia were available should have minimised problems encountered in earlier investigations. Furthermore, because of the lower than expected risk of lung cancer in this population, potential confounding influences, particularly smoking, must be considered. However, since smoking has been associated with stomach cancer risk in some studies (Correa *et al.*, 1985; Hirayama, 1981; Kahn, 1966), an under-representation of smokers in the pernicious anaemia cohort would have caused an attenuation in the observed risks. Finally, follow-up for cancer relied on information in VA medical records. If patients with pernicious anaemia were followed more closely than the general VA population, cancer risks could have been overestimated. Thus, it was reassuring to find that the cancer elevations were confined to specific sites hypothesised *a priori* to be of interest.

Despite the limitations of this study, our findings are consistent with previous estimates that patients with pernicious anaemia have a risk of stomach cancer about three times that of the general population. The precursor lesion appears to be an atrophic gastropathy that leads to pernicious anaemia, and results at least partly from autoimmune mechanisms (Burnier *et al.*, 1976). However, the attributable risk of stomach cancer among pernicious anaemia patients is relatively small. In comparison to persons without pernicious anaemia, the excess risk of stomach cancer in this study was only 61.1 per 100,000 per year. This suggests that the cost-effectiveness of periodic long-term screening (e.g. endoscopy) should be reassessed for pernicious anaemia, with an aim to identifying patients most likely to benefit because of advancing precursor lesions.

### References

BERKSON, J., COMFORT, M.W. & BUTT, H.R. (1956). Occurrence of gastric cancer in persons with achlorhydria and with pernicious anemia. *Proc. Staff Meet. Mayo Clin.*, **31**, 583.

BLACKBURN, E.K., CALLENDER, S.T., DACIE, J.V. and 8 others (1968). Possible association between pernicious anaemia and leukaemia: a prospective study of 1,625 patients with a note on the very high incidence of stomach cancer. *Int. J. Cancer*, **3**, 163.

BLATTNER, W.A., BLAIR, A. & MASON, T.J. (1981). Multiple myeloma in the United States, 1950–1975. *Cancer*, **48**, 2547.

BLOT, W.J., McLAUGHLIN, J.K., WINN, D.M. and 7 others (1988). Smoking and drinking in relation to oral and pharyngeal cancer. *Cancer Res.*, **48**, 3282.

BURNIER, E., ZWAHLEN, A. & CRUCHAUD, A. (1976). Non-malignant monoclonal immuno-globulinemia, pernicious anemia and gastric carcinoma. A model of immunologic dysfunction. Report of two cases and review of the literature. *Am. J. Med.*, **60**, 1019.

CHANARIN, I. (1969). *The Megaloblastic Anaemias*, p. 457. Blackwell Scientific: Oxford.

CORCINO, J.J., ZALUSKY, R., GREENBERG, M. & HERBERT, V. (1971). Coexistence of pernicious anaemia and chronic myeloid leukaemia: an experiment of nature involving vitamin $B_{12}$ metabolism. *Br. J. Haematol.*, **20**, 511.

CORREA, P., FONTHAM, E., PICKLE, L.W., CHEN, V., LIN, Y.P. & HAENSZEL, W. (1985). Dietary determinants of gastric cancer in south Louisiana inhabitants. *JNCI*, **75**, 645.

DOUGLAS, A.S. & RIFKIND, B.M. (1964). Megaloblastic anaemia in association with polycythemia vera. *Scott. Med. J.*, **9**, 469.

ELSBORG, L. & MOSBECH, H. (1979). Pernicious anaemia as a risk factor in gastric cancer. *Acta Med. Scand.*, **206**, 315.

ENGEL, A.G. & STICKNEY, J.M. (1962). Pernicious anemia and polycythemia vera in one patient. *Arch. Intern. Med.*, **109**, 168.

ERIKSSON, S., CLASE, L. & MOQUIST-OLSSON, I. (1981). Pernicious anemia as a risk factor in gastric cancer. The extent of the problem. *Acta Med. Scand.*, **210**, 481.

FRASER, K.J. (1969). Multiple myeloma and pernicious anaemia. *Med. J. Aust.*, **i**, 298.

GEOGHEGAN, G.E. & PAGE, W.F. (1984). *Health Characteristics of Male Veterans and Nonveterans, Health Interview Surveys, 1971–1981*. Office of Information Management and Statistics, Statistical Policy and Research Service: Washington, DC.

GREENE, M.H., YOUNG, T.I. & CLARK, W.H. JR (1981). Malignant melanoma in renal transplant recipients. *Lancet*, **i**, 1196.

HIRAYAMA, T. (1981). Proportion of cancer attributable to occupation obtained from a census population-based, large cohort study in Japan. *Banbury Rep.*, **9**, 631.

HITCHCOCK, C.R., SULLIVAN, W.A. & WANGENSTEEN, O.H. (1955). The value of achlorhydria as a screening test for gastric cancer: a 10-year report. *Gastroenterology*, **29**, 621.

KAHN, H.A. (1966). The Dorn Study of Smoking and Mortality Among U.S. Veterans: report of eight and one-half years of observation. *Natl Cancer Inst. Monogr.*, **19**, 1.

KAPLAN, H.S. & RIGLER, L.G. (1945). Pernicious anemia and carcinoma of the stomach-autopsy studies concerning their interrelationship. *Am J. Med. Sci.*, **209**, 339.

LARSSON, S.O. (1962). Myeloma and pernicious anaemia. *Acta Med. Scand.*, **172**, 195.

LIDDELL, F.D.K. (1984). Simple exact analysis of the standardized mortality ratio. *J. Epidemiol. Comm. Hlth*, **38**, 85.

LOWE, W.C. (1976). Hypogammaglobulinemia, pernicious anemia with carcinoma of urinary bladder and lung. *NY St. J. Med.*, **76**, 926.

McLAUGHLIN, J.K., GRIDLEY, G., BLOCK, G. and 9 others (1988). Dietary factors in oral and pharyngeal cancers. *JNCI*, **80**, 1237.

MASON, T.J., FRAUMENI, J.F., JR, HOOVER, R. & BLOT, W.J. (1981). *An Atlas of Mortality from Selected Diseases*, NIH publication no. 81-2397. US Govt. Printing Office: Washington, DC.

MITCHELL, K., FERGUSON, M.M., LUCIE, N.P. & MACDONALD, D.G. (1986). Epithelial dysplasia in the oral mucosa associated with pernicious anaemia. *Br. Dent. J.*, **161**, 259.

NIELSEN, V.G. & JENSEN, M.K. (1970). Pernicious anaemia and acute leukaemia: a case report with cytogenetic studies. *Scand. J. Haematol.*, **7**, 26.

RIDDLE, E.M. & DAVIDSON, R.J.L. (1968). Coexistence of pernicious anemia and acute erythremic myelosis. *J. Clin. Pathol.*, **21**, 590.

US PUBLIC HEALTH SERVICE (1980). *The International Classification of Diseases 9th Revision Clinical Modification*. Commission on Professional and Hospital Activities: Ann Arbor, MC.

VOGELSANG, G.B. & SPIVAK, J.L. (1984). Unusual case of acute leukemia: coexisting acute leukemia and pernicious anemia. *Am. J. Med.*, **76**, 1144.

ZAMCHECK, N., GRABLE, E., LEY, A. & NORMAN, L. (1955). Occurrence of gastric cancer among patients with pernicious anemia at the Boston City Hospital. *N. Engl. J. Med.*, **252**, 1103.

ZARAFONETIS, C.J.D., OVERMAN, R.L. & MOLTHAN, L. (1975). Unique sequence of pernicious anemia, polycythemia, and acute leukemia. *Blood*, **12**, 1011.


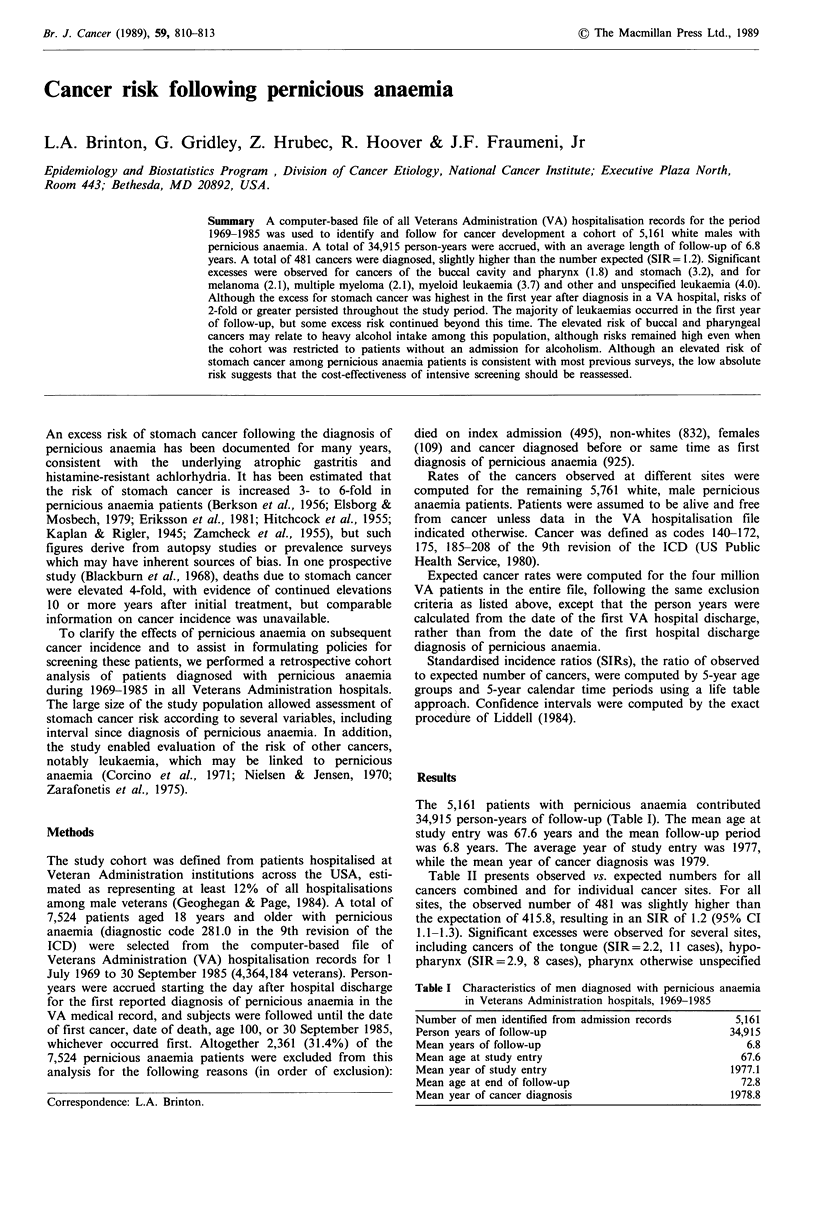

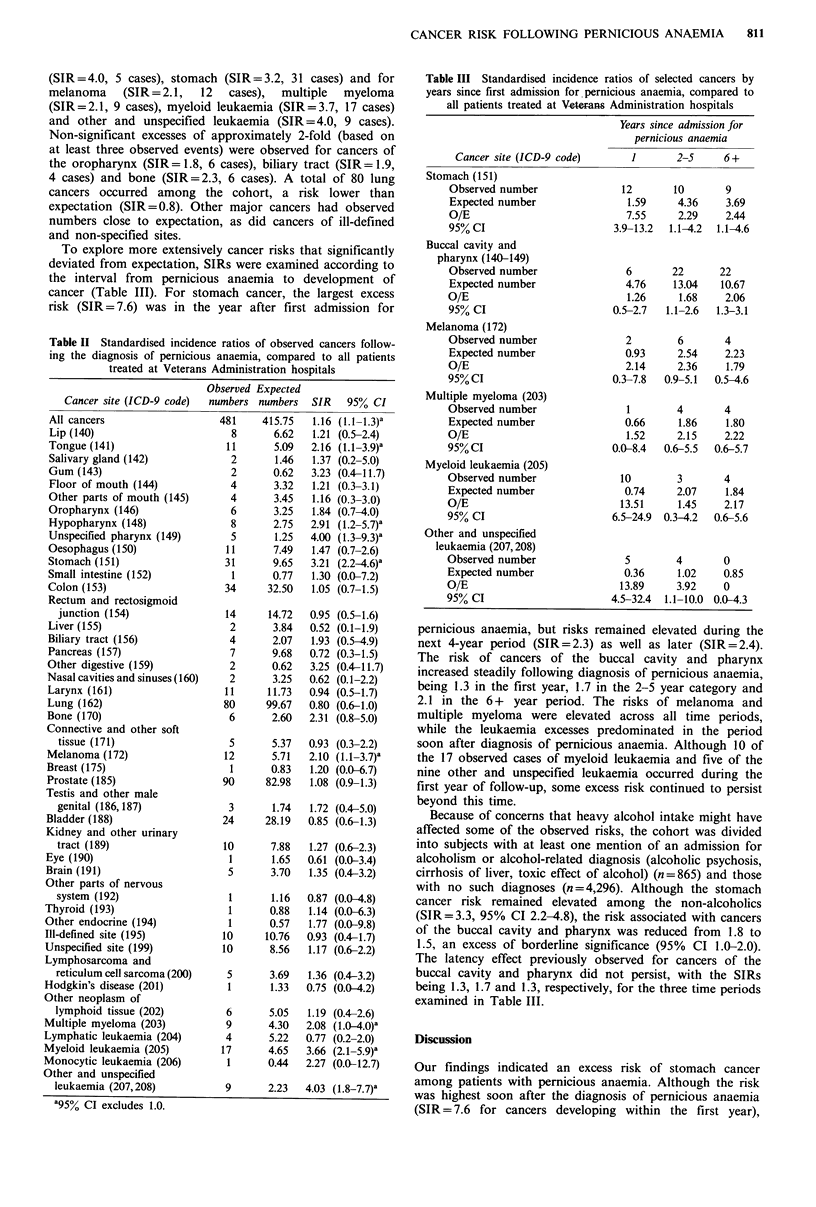

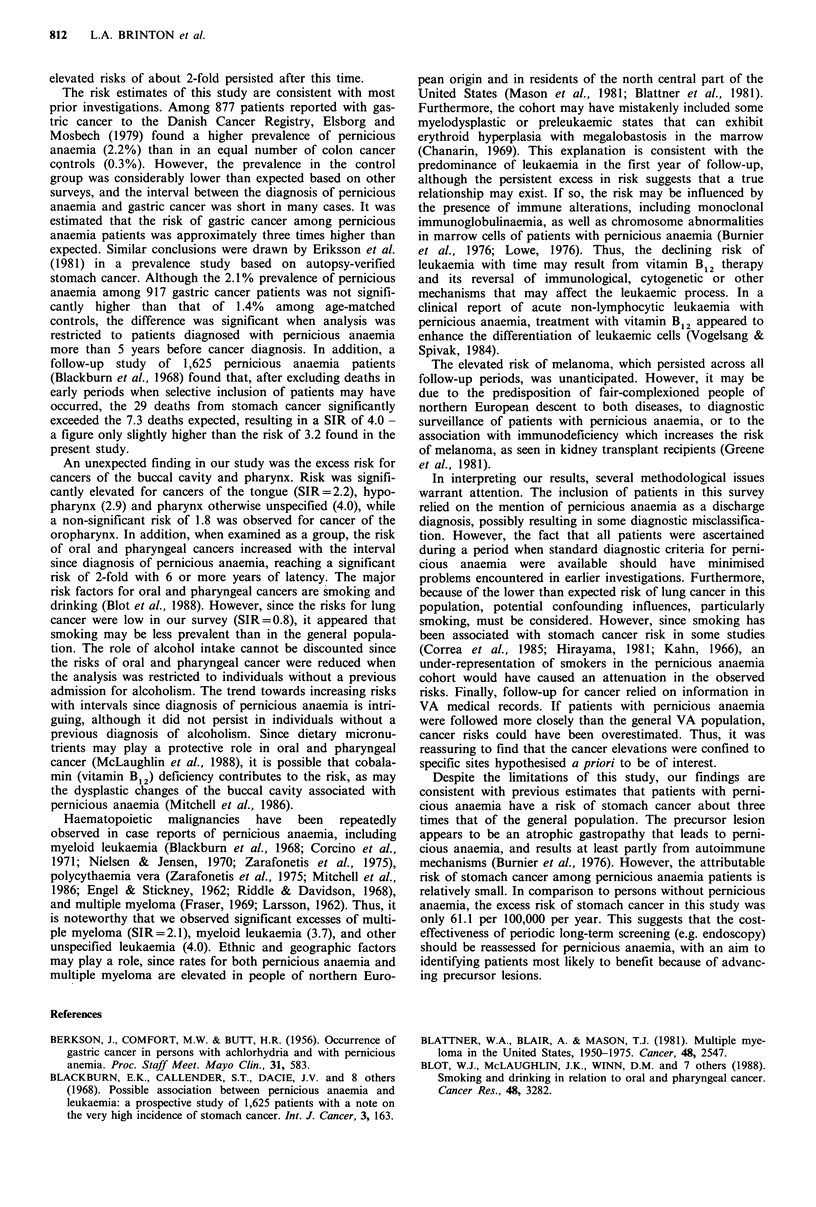

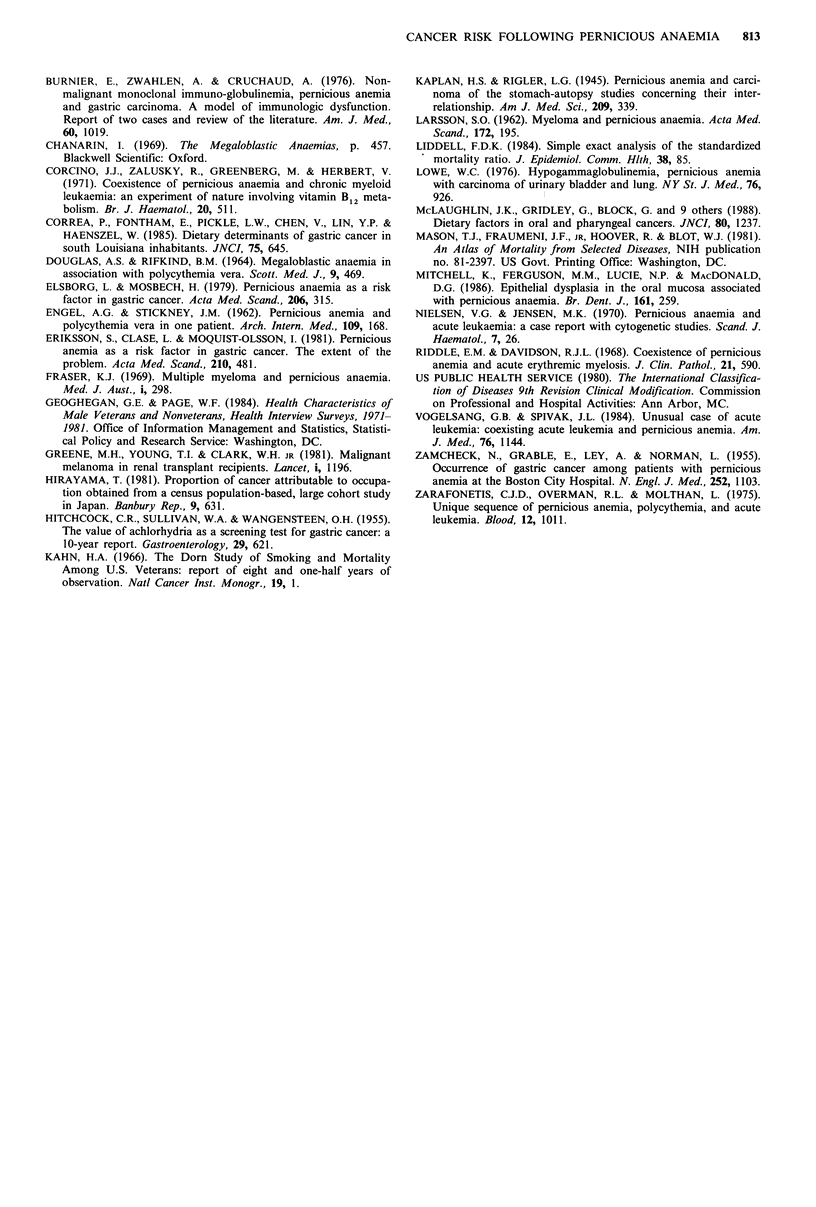


## References

[OCR_00604] BERKSON J., BUTT H. R., COMFORT M. W. (1956). Occurrence of gastric cancer in persons with achlorhydria and with pernicious anemia.. Proc Staff Meet Mayo Clin.

[OCR_00609] Blackburn E. K., Callender S. T., Dacie J. V., Doll R., Girdwood R. H., Mollin D. L., Saracci R., Stafford J. L., Thompson R. B., Varadi S. (1968). Possible association between pernicious anaemia and leukaemia: a prospective study of 1,625 patients with a note on the very high incidence of stomach cancer.. Int J Cancer.

[OCR_00615] Blattner W. A., Blair A., Mason T. J. (1981). Multiple myeloma in the United States, 1950--1975.. Cancer.

[OCR_00619] Blot W. J., McLaughlin J. K., Winn D. M., Austin D. F., Greenberg R. S., Preston-Martin S., Bernstein L., Schoenberg J. B., Stemhagen A., Fraumeni J. F. (1988). Smoking and drinking in relation to oral and pharyngeal cancer.. Cancer Res.

[OCR_00626] Burnier E., Zwahlen A., Cruchaud A. (1976). Nonmalignant monoclonal immunoglobulinemia, pernicious anemia and gastric carcinoma. A model of immunologic dysfunction. Report of two cases and review of the literature.. Am J Med.

[OCR_00637] Corcino J. J., Zalusky R., Greenberg M., Herbert V. (1971). Coexistence of pernicious anaemia and chronic myeloid leukaemia: an experiment of nature involving vitamin B12 metabolism.. Br J Haematol.

[OCR_00643] Correa P., Fontham E., Pickle L. W., Chen V., Lin Y. P., Haenszel W. (1985). Dietary determinants of gastric cancer in south Louisiana inhabitants.. J Natl Cancer Inst.

[OCR_00648] DOUGLAS A. S., RIFKIND B. M. (1964). MEGALOBLASTIC ANAEMIA IN ASSOCIATION WITH POLYCYTHAEMIA VERA.. Scott Med J.

[OCR_00652] Elsborg L., Mosbech J. (1979). Pernicious anaemia as a risk factor in gastric cancer.. Acta Med Scand.

[OCR_00659] Eriksson S., Clase L., Moquist-Olsson I. (1981). Pernicious anemia as a risk factor in gastric cancer. The extent of the problem.. Acta Med Scand.

[OCR_00664] Fraser K. J. (1969). Multiple myeloma and pernicious anaemia.. Med J Aust.

[OCR_00674] Greene M. H., Young T. I., Clark W. H. (1981). Malignant melanoma in renal-transplant recipients.. Lancet.

[OCR_00683] HITCHCOCK C. R., SULLIVAN W. A., WANGENSTEEN O. H. (1955). The value of achlorhydria as a screening test for gastric cancer.. Gastroenterology.

[OCR_00688] Kahn H. A. (1966). The Dorn study of smoking and mortality among U.S. veterans: report on eight and one-half years of observation.. Natl Cancer Inst Monogr.

[OCR_00698] LARSSON S. O. (1962). Myeloma and pernicious anaemia.. Acta Med Scand.

[OCR_00702] Liddell F. D. (1984). Simple exact analysis of the standardised mortality ratio.. J Epidemiol Community Health.

[OCR_00706] Lowe W. C. (1976). Hypogammaglobulinemia, pernicious anemia: with carcinomas of urinary bladder and lung.. N Y State J Med.

[OCR_00711] McLaughlin J. K., Gridley G., Block G., Winn D. M., Preston-Martin S., Schoenberg J. B., Greenberg R. S., Stemhagen A., Austin D. F., Ershow A. G. (1988). Dietary factors in oral and pharyngeal cancer.. J Natl Cancer Inst.

[OCR_00719] Mitchell K., Ferguson M. M., Lucie N. P., MacDonald D. G. (1986). Epithelial dysplasia in the oral mucosa associated with pernicious anaemia.. Br Dent J.

[OCR_00724] Nielsen V. G., Jensen M. K. (1970). Pernicious anaemia and acute leukaemia. A case report with cytogenetic studies.. Scand J Haematol.

[OCR_00729] Riddell E. M., Davidson R. J. (1968). Coexistance of pernicious anaemia and acute erythraemic myelosis.. J Clin Pathol.

[OCR_00737] Vogelsang G. B., Spivak J. L. (1984). Unusual case of acute leukemia. Coexisting acute leukemia and pernicious anemia.. Am J Med.

[OCR_00742] ZAMCHECK N., GRABLE E., LEY A., NORMAN L. (1955). Occurrence of gastric cancer among patients with pernicious anemia at the Boston City Hospital.. N Engl J Med.

[OCR_00746] ZARAFONETIS C. J., OVERMAN R. L., MOLTHAN L. (1957). Unique sequence of pernicious anemia, polycythemia, and acute leukemia.. Blood.

